# Supplementary material for: Patterns of Intron Gain and Loss in Fungi
Source: PLoS Biol. 2004 Nov 30;2(12):e422. doi: 10.1371/journal.pbio.0020422 (PMC532390; doi:10.1371/journal.pbio.0020422)
Supplement: Table S1 — Also available at http://genes.mit.edu/NielsenEtAl/. (4.3 MB ZIP). [file pbio.0020422.st001.zip › NielsenEtAl/html/239.html]

AN6536.1.NCU01300.1.MG07528.1.FG06093.1


```
 CLUSTAL W (1.82) Multiple Sequence Alignments - Introns Inserted


Sequence 1: NCU01300.1	229 aa
Sequence 2: MG07528.1	225 aa
Sequence 3: FG06093.1	227 aa
Sequence 4: AN6536.1	244 aa
Alignment Length: 249 aa
Number Identitical Residues: 116 aa
Alignment Score (without introns) 5667


MG07528.1 	----MASPRWAALARDTNETKVKLALSIDGGDLPPDTHPS-----------------LLE
NCU01300.1	MSTEQPAPRWAAFARDTNETKIQIALNLDGGAFPPETDAR-----------------LNV
FG06093.1 	-MASSQPTRWAALARDTNETKIQLALNLDGGDFPPDTDSR-----------------LLD
AN6536.1  	---MPLPARTASLSRNTNETKIQVSLSLDGGVLPPYEPSSHFPVPTDPLEAEAAKKGIVP
          	      ..* *:::*:*****::::*.:*** :**   .   . .:.. .:.::...:  

MG07528.1 	AAASGHASQSSSSQKISINTGIGFLDHMLHAFAKHAGFSLLLTCQGDLHI1DDHHTAEDV
NCU01300.1	SIADGHATQASKSQTISVNTGIGFLDHMLHALAKHAGWSLALACKGDLHI1DDHHTAEDV
FG06093.1 	NDDS-HASQASKSQKIAVNTGIGFLDHMLHALAKHAGWSLALNCKGDLHI1DDHHTAEDV
AN6536.1  	NKDAAHATQFTPTQQITISTGIGFLDHMLHALAKHSGWSLAIRAKGDLYI1DDHHTTEDT
          	    .**:* : :* *::.************:***:*:** : .:***:* *****:**.

MG07528.1 	CIALGDAFKTALGSAAGIARFGFAYCPLDEALSRAVVDVSNRPFAVVELGLRREKIGDLS
NCU01300.1	CIALGYAFAKALGTPTGLARFGSAYCPLDEALSRAVVDLSNRPFAVVDLGLKREKIGDLS
FG06093.1 	CIALGYAFAQALGTPAGLARFGYAYAPLDEALSRAVIDLSNRPYAVVDLGLKREWLGQLS
AN6536.1  	FLALGTAFTTALGARQSLARFGRGDAPLDEALSWAVIDLSSRPWAVINIGFKREKIGDLS
          	 :*** **  ***:  .:**** . .******* **:*:*.**:**:::*::** :*:**

MG07528.1 	CEMIPHCIMSFATAARLTIHVDCIRGENDHHRAESAFKALAVAVRTAVG-KVAGRE--GE
NCU01300.1	TEMIPHCIHSFAGAARITVHVDCLRGDNDHHRAESAFKALAVATRQATS-RVAGRE--GE
FG06093.1 	TEMVPHCLQSFAQGARVTLHVHCLHGDNDHHRAESAFKALAIAVKAATT-RIPGKE--GE
AN6536.1  	TEMITHGLQSFAQAAGVTLHIGCTYGDNDHHRAESAFKALAVAIRAACARRVEGEVGAGD
          	 **:.* : *** .* :*:*: *  *:**************:* : *   :: *. .:*:

MG07528.1 	VPSTKGTLSV
NCU01300.1	VPSTKGTLSV
FG06093.1 	VPSTKGTLSA
AN6536.1  	VVSTKGVL--
          	* ****.*
```
